# Increasing the biocompatibility of graphene-based hybrid nanostructures with glycopolymer

**DOI:** 10.55730/1300-0527.3316

**Published:** 2021-11-29

**Authors:** Aydan DAĞ, Pınar Sinem OMURTAG ÖZGEN, Belma ZENGİN KURT, Zehra DURMUŞ

**Affiliations:** 1 Department of Pharmaceutical Chemistry, Faculty of Pharmacy, Bezmialem Vakıf University, İstanbul, Turkey; 2 Drug Application and Research Center, Bezmialem Vakıf University, İstanbul, Turkey; 3 Department of Analytical Chemistry, School of Pharmacy, İstanbul Medipol University, İstanbul, Turkey; 4 Centre for Innovation Competence (ZIK) SiLi-nano, Martin Luther University Halle-Wittenberg, Halle (Salle), Germany

**Keywords:** Nanographene, glycopolymer, π-π stacking, lectin, concanavalin A

## Abstract

Hybrid nanostructures decorated with glycopolymers are appropriate for biomedical applications. In this paper, the results are obtained from nanographene (NG) decorated with glycoblock copolymer to increase their potential in use in therapies and in examining lectin interactions. A pyren-1-ylmethyl 4-cyano-4-((phenylcarbonothioyl)thio)pentanoate (CPADB-py) chain transfer agent was used in the synthesis of methyl methacrylate glycoblock copolymers (P2 and P3) by reversible addition-fragmentation chain transfer (RAFT) polymerization to adhere the polymer to the nanographene surface. Hybrid nanographenes (NG-1 and NG-2) were obtained by the non-covalent interaction of deprotected P2 and P3 with different fructopyranose groups (3-*O*-methacryloyl-1,2:4,5-di-*O*-isopropylidene-*β*-D-fructopyranose and 1-*O*-methacryloyl-2,3:4,5-di-*O*-isopropylidene-*β*-D-fructopyranose) in their backbones. Images obtained from transmission electron microscopy (TEM) of NG-1 and NG-2 show that glycoblock copolymer coating was performed homogeneously. Moreover, thermal gravimetric analysis (TGA) also confirmed a glycoblock copolymer coating of NG-1 and NG-2 by weight loss of 41% and 31%, respectively. In the last step of the study, the binding ability of glycoblock copolymers (P2-hyd and P3-hyd) with concanavalin A (ConA) lectin was investigated by a turbidimetric assay. Promising results were obtained from P3-hyd for the ConA interactions. Hence, this study may open a new avenue in the design of new multifunctional glyconanomaterials that show favorable binding properties with lectins.

## 1. Introduction

The use of hybrid graphene structures obtained by surface modification with polymers in biomedical applications has increased in recent years. Nanographene modification by a biocompatible polymer is frequently used in therapy and diagnosis. Glycopolymers, which are in the class of biocompatible polymers, are recognized by the cell through their interaction with lectins on the cell surface. The research on theranostic applications of hybrid nanostructures has increased in recent years due to their inherent properties and unique abilities in the diagnosis and treatment of diseases. Particularly, nanotechnology-based systems play a key role in designing hybrid nanostructures with specific functional properties. The advantages of hybrid nanostructures are certainly because of their nanoscale size and large specific surface areas that can be functionalized with targeting compounds and diagnostic/therapeutic moieties. New hybrid nanostructures may be obtained, or they may be placed in biomaterials to obtain new functionalities. Hence, multifunctional hybrid nanostructures are desirable for many biomedical applications such as diagnosis [1,2], controlled drug delivery [3], photothermal/photodynamic/photoacoustic therapy [4], and hyperthermia [5,6].

Studies on graphene and its applications have been carried out following the discovery of graphene in 2004 [7]. Graphene, a flat monolayer of *sp*^2^-bonded carbon atoms tightly packed into a two-dimensional honeycomb lattice, is considered the thinnest and lightest *sp*^2^ carbon nanomaterial in the universe. It exhibits fascinating physicochemical, thermal, optical, mechanical, and biological properties such as fast electron mobility, high current density, high mechanical strength, excellent thermal conductivity, and ultra-larger surface area. These characteristics of graphene-based nanosheets such as graphene (G), graphene oxides (GO), and reduced graphene oxides (rGO) make these nanomaterials ideal components for nanoelectronics, nanodevices, and nanocomposites. Functionalizing graphene-based materials or synthesizing their hybrid structures provide an even greater application opportunity to adapt their properties while enabling advanced or new features to be used in a wider range of applications. Additionally, the planar structure of graphene-based nanosheets ensures effective loading of a variety of substances of desired biomolecules such as enzymes and proteins, either through passive adsorption or by covalent bonding to the reactive groups of biomolecules [8]. Lately, great attempts have also been undertaken for discovering the potential usage of graphene and graphene-based materials as nanocarriers in biomedicine and biological applications as nanocarriers [9–11].

Due to its excellent structural properties, the incorporation of graphene into polymer composite/hybrid structures has led to numerous studies on the production of hybrids/composites with superior physicochemical, mechanical, thermal, and biological properties. However, the interfacial interactions and dispersibility of graphene with polymer matrices directly affect the properties of the desired nanocomposite. Therefore, graphene’s surface functionalization is important for both solubility and usefulness. Surface modification of graphene can be done by both covalent and noncovalent means to obtain key advantages in biomedical applications by functionalization as delivery platforms for a variety of molecules relevant to therapy and diagnosis.

A noncovalent surface modification of graphene consists of a variety of conjugations without disturbing the extended p-conjugation of the polyaromatic surface of graphene such as π-π stacking and van der Waals interactions, physical attachment, electrostatic adsorption, or hydrogen bonding [12].

The aromatic lattice of graphene oxide consists of *sp*^2^-hybridized carbons and its basal plane, and the edges consist mainly of *sp*^3^-hybridized carbons with hydroxyl (−OH) and epoxide groups. While the interaction between electronegative atoms of molecules/compounds and functional groups of graphene oxide can be achieved by hydrogen bonding, the existence of the ionizable carboxylic acid groups on the edges of GO allows for electrostatic interactions. Besides the existence of these functional groups, the main plane of graphene oxide is basically composed of polyaromatic networks that enable molecules to bond through π-π stacking and/or hydrophobic interactions [13]. When rGO is compared to GO, it is more hydrophobic due to having fewer oxygen-containing functional groups. In reduced GO, a significant number of oxygen functional groups are removed from the structure by chemical methods, resulting in a more uniform and spaced monolayer. The removal of these groups results in an interaction capacity of the structure through hydrogen bonding or electrostatic interactions less than that of graphene oxide. By contrast, π-π stacking and hydrophobic interactions with chemical structures are more dominant in rGO. Thus, in terms of hydrophobicity, rGO is higher than GO but less than graphene layers [14]. Nano graphene, smaller graphene flakes as single-layered graphene, has a higher surface area, which is associated with high reactivity. Because the number of layers decreases towards multilayer graphene to single-layered graphene, the surface area increases [15].

The processability and dispersibility of the modified nanographene platelets can be utilized for the creation of graphene-based new nanomaterials for advanced biomedical applications.

The interactions between saccharides and proteins play important roles in many biological events, including cell-cell recognition, cell differentiation, and cell−cell adhesion. In addition, the cells are infected by various pathogens, such as viruses, bacteria, and toxins, through interactions of saccharides with proteins. A notable example can be given for the interactions between Sars-CoV-2 virus and sialyl oligosaccharides. It is thought that the spike glycoprotein undergoes a conformational change to bind to a protein termed ACE2, which is the center target and is common in cells. Thus, resembling microorganisms (viruses and bacteria) involves the generation of nanoscale carriers with a set of functional groups on their surface containing proteins and glycoproteins.

Polysaccharides have wide molecular weight distributions and randomly branched structures. Many synthetic attempts have been made to create similar structures in mimicking the roles of polysaccharides. Glycopolymers are synthetic polymers carrying carbohydrate functional groups. Glycopolymers, which are in the class of biocompatible polymers, are recognized by the cell through their interaction with lectins on the cell surface.

Significant advances have been revealed in synthetic glycopolymers used in biological systems such as molecular recognition and medical applications by mimicking the roles of polysaccharides. These include glycodendrimers and linear glycopolymers such as micelles, vesicles, and micro/nanoparticles. Linear, hyperbranched and dendrimeric glycopolymers have been widely used to understand the interaction between glycopolymers and lectins [16–19]. Glycopolymers, glycoparticles, or glycopolymer decorated surfaces show a nonlinear increase in binding affinity due to the cluster glycoside effect [20,21].

Concanavalin A (ConA) is a plant lectin that has revealed extensive information about interactions between saccharides and protein. One of the most important parameters for determining how to achieve good binding is identifying the best fit between the ligand and the binding site. Many studies have demonstrated that the density of sugar residues and interactions in terms of multiple binding plays a vital role in elaborating the saccharide protein interactions [22,23]. Besides macromolecular architecture, the chain structure of glycopolymers, such as block, statistical, random, alternating, and gradient, has been shown to have a key influence on their properties [24].

Increasing the therapeutic potential of these nanostructures and examining their lectin interactions are important for more refined applications in cell biology.

**Figure 1:**
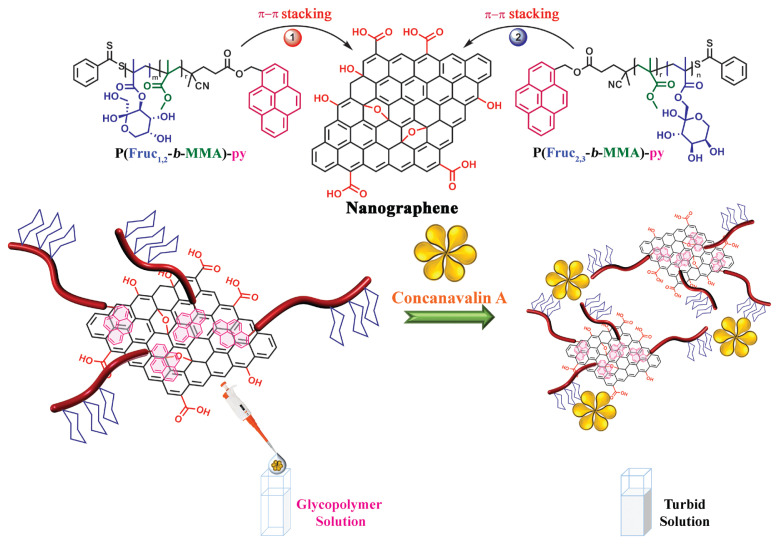
Schematic representation for glycoblock copolymer attachment into nanographene (NG).

This study in which we showed the synthesis and structural analysis of glycoblock copolymer decorated nanographene (NG) aims to present new perspectives for the applications of functionalized carbon-based materials in nanomedicine, by expanding knowledge of their interactions with various cell surfaces, pathogens and proteins (Figure 1) [25].

Hence, two different sugar moieties of (3-*O*-methacryloyl-1,2:4,5-di-*O*-isopropylidene-*β*-D-fructopyranose (*ipr*Fruc_1,2_) and 1-*O*-methacryloyl-2,3:4,5-di-*O*-isopropylidene-*β*-D-fructopyranose (*ipr*Fruc_2,3_)) containing glycoblock copolymer were first synthesized via the RAFT polymerization technique. Then, isopropylidene groups of P(*ipr*Fruc_1,2_MA-*b*-MMA)-py (P2) and P(*ipr*Fruc_2,3_MA-*b*-MMA)-py (P3) diblock glycopolymers were removed via hydrolysis reaction. After successful characterization of the deprotected diblock glycopolymers (P2-hyd and P3-hyd), hybrid nanographene (NG-1 and NG-2) was obtained by noncovalent interaction of P2-hyd and P3-hyd. The interactions of the P2-hyd and P3-hyd glycopolymers with ConA was performed using time-dependent UV measurements and direct visualization of the turbidity of the solutions.

## 2. Materials and methods

Pyren-1-ylmethyl 4-cyano-4-((phenylcarbonothioyl)thio) pentanoate (CPADB-py) [26], 3-*O*-methacryloyl-1,2:4,5-di-*O*-isopropylidene-*β*-D-fructopyranose (*ipr*Fruc_1,2_MA) [27] and 1-*O*-methacryloyl-2,3:4,5-di-*O*-isopropylidene-*β*-D-fructopyranose (*ipr*Fruc_2,3_MA) [27] were prepared according to procedures of given references and analytical data are reported in Supporting Information (Figure S1–S6).

### 2.1. Synthesis of Pyrene End-Capped Poly(Methyl methacrylate) as Macro-RAFT Agent (PMMA-py, P1)

**Figure 2:**
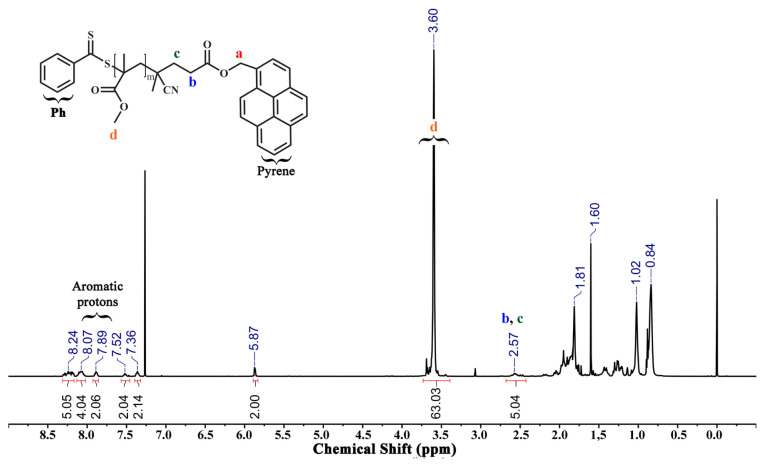
^1^H NMR spectrum of PMMA_21_-py in (P1) CDCl_3_.

Methyl methacrylate (2 mL, 18.70 mmol), azobisisobutyronitrile (AIBN) (7.67 mg, 0.046 mmol) and CPADB-py as chain transfer agent (184 mg, 0.374 mmol) were added into a round bottom flask and dissolved in 4.0 mL acetonitrile (ACN). The polymerization was subsequently placed in an oil bath at 70 °C. The polymerization reaction was quenched after 6.5 h via a rapid cooling process and opened to air. Then, the mixture was precipitated in 10-fold excess of hexane two times to remove the unreacted monomer [28]. The purified polymer was dried under vacuum for 24 h (yield: 0.598 g, 32%). See supporting information Scheme S1 and Figure 2.

### 2.2. General Route for Synthesis of Glycoblock Polymers (P(*ipr*Fruc_1,2_MA-*b*-MMA)-py and P(*ipr*Fruc_2,3_MA-*b*-MMA)-py, P2 and P3)

**Figure 3:**
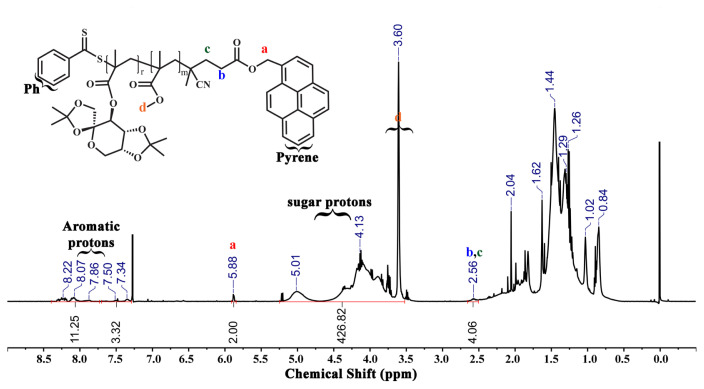
^1^H NMR spectrum of P(*ipr*Fruc_1,2_MA_52_-*b*-MMA_21_)-py (P2) copolymer in CDCl_3_.

**Figure 4:**
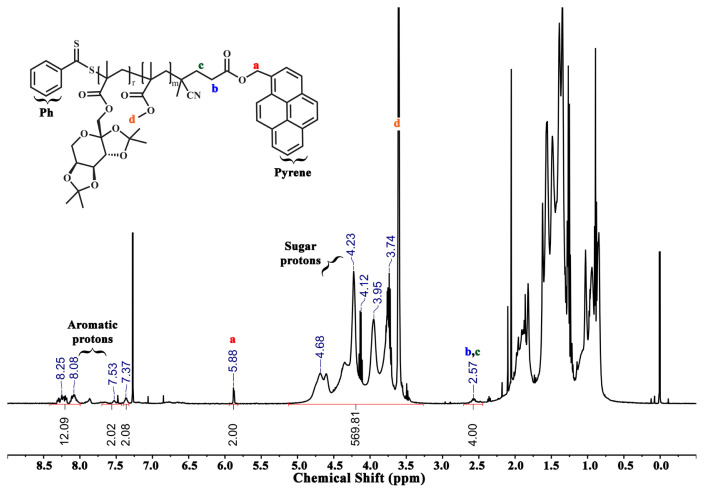
^1^H NMR spectrum of P(*ipr*Fruc_2,3_MA_72_-*b*-MMA_21_)-py (P3) copolymer in CDCl_3_.

PMMA-py (P1) was applied as a Macro-RAFT agent. Initially, PMMA-py (125 mg, 0.048 mmol, *M*_n,NMR_ = 2.60 kDa), *ipr*Fruc_1,2_MA (1.105 g, 3.365 mmol), AIBN (1.60 mg, 9.61×10^−3^ mmol) and ACN (2.0 mL) were added into a round-bottom flask. The mixture was bubbled with nitrogen for 45 min and stirred in an oil bath at 70 °C for 8.5 h. To remove the unreacted monomer and purify, the glycoblock copolymer, P(*ipr*Fruc_1,2_MA-*b*-MMA)-py (P2) was precipitated twice in hexane. Then P2 was dried under vacuum overnight at 25 °C. The yield of P2 was 0.655 g. The same procedure was employed by using a 1.105 g *ipr*Fruc_2,3_MA monomer to synthesize P(*ipr*Fruc_2,3_MA-*b*-MMA)-py (P3) [28]. The yield of P(*ipr*Fruc_2,3_MA-*b*-MMA)-py was 0.686 g. Nuclear magnetic resonance (^1^H NMR) and gel permeation chromatography (GPC) measurements were carried out to characterize both glycoblock copolymers (Figures 3, 4, and S7).

### 2.3. Typical Procedure for Deprotection of Glycoblock Copolymers (P2-hyd and P3-hyd)

**Figure 5:**
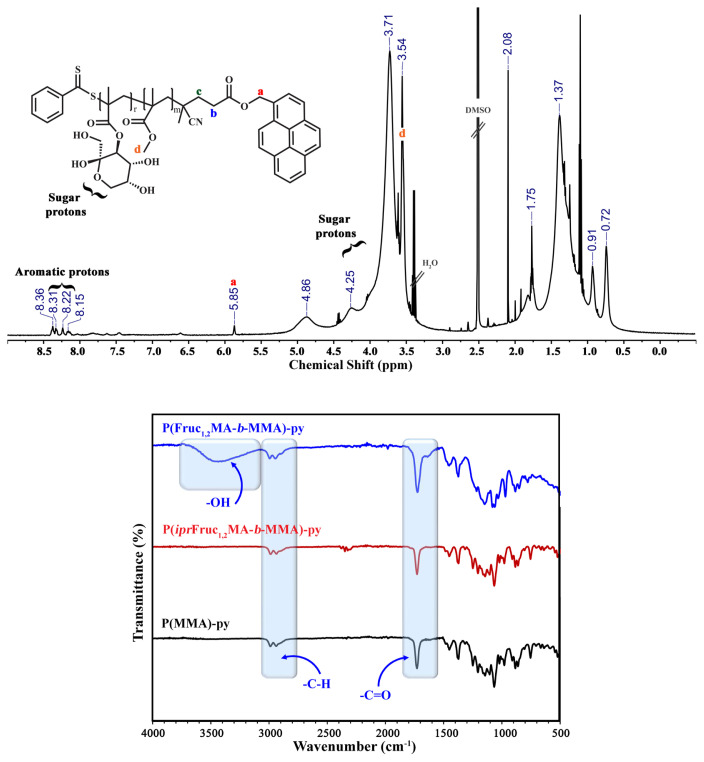
**A)** ^1^H NMR spectrum of P(Fruc_1,2_MA_52_-*b*-MMA_21_)-py (P2-hyd) copolymer in DMSO-*d*_6_ **B)** IR spectra overlay of PMMA_21_-py (P1), P(*ipr*Fruc_1,2_MA_52_-*b-*MMA_21_)-py (P2) and P(Fruc_1,2_MA_52_-*b-*MMA_21_)-py (P2-hyd).

**Figure 6:**
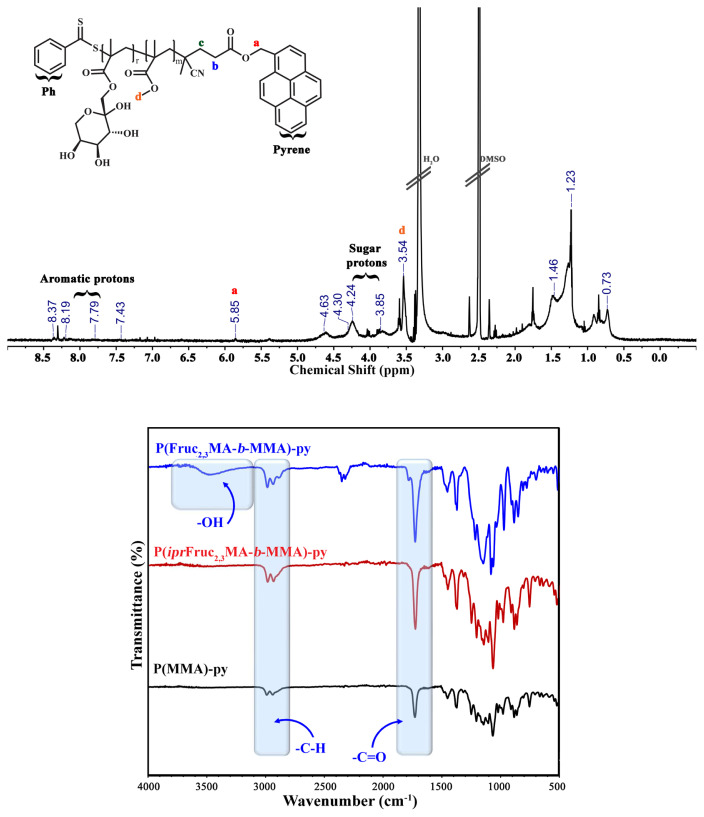
**A)** ^1^H NMR spectrum of P(Fruc_2,3_MA_72_-*b*-MMA_21_)-py (P3-hyd) copolymer in DMSO-*d*_6_ **B)** IR spectra overlay of PMMA_21_-py (P1), P(*ipr*Fruc_2,3_MA_72_-*b-*MMA_21_)-py (P3) and P(Fruc_2,3_MA_72_-*b-*MMA_21_)-py (P3-hyd).

The deprotection of the isopropylidene groups of glycoblock copolymers was performed under acidic conditions. The polymer (650 mg) was dissolved in 10 mL of CHCl_3,_ and then 2 mL of TFA/H_2_O (9:1, v/v) solution mixture was added in a round-bottom flask. After overnight stirring at room temperature, the glycoblock copolymer was precipitated twice in diethyl ether, and then the crude polymer was dialyzed against Milli-Q water for two days (MWCO 3500). The obtained polymer was then freeze-dried. The resulting deprotected polymers (P(Fruc_1,2_MA-*b*-MMA)-py; P2-hyd and P(Fruc_2,3_MA-*b*-MMA)-py; P3-hyd) were characterized by following the removal of isopropylidene signals by ^1^H NMR and FT-IR (Figure 5 and 6). The yield for P2-hyd was found as 0.631 g and 0.401 g for P3-hyd [27].

### 2.4. Synthesis of nanographene

Initially, oxidation of expandite graphite powders was performed according to the modified Hummers procedure [29]. The details of the oxidation and reduction processes, the structural characterization, and the preparation of reduced expanded graphene oxide (called graphene) were described previously [30]. Secondly, to provide smaller graphene flakes, yielded sample was re-dispersed in distilled water and placed in an ultrasonic homogenizer for 6 h. The sonication was followed by a centrifugation step at 8000 rpm for 30 min, obtaining the separation of two different phases. The upper phase corresponds to nanographene particles and was yielded for next step use.

### 2.5. Typical protocol for functionalization of nanographene with pyrene end-capped glycoblock copolymers via π-π stacking (NG-1 and NG-2)

**Figure 7:**
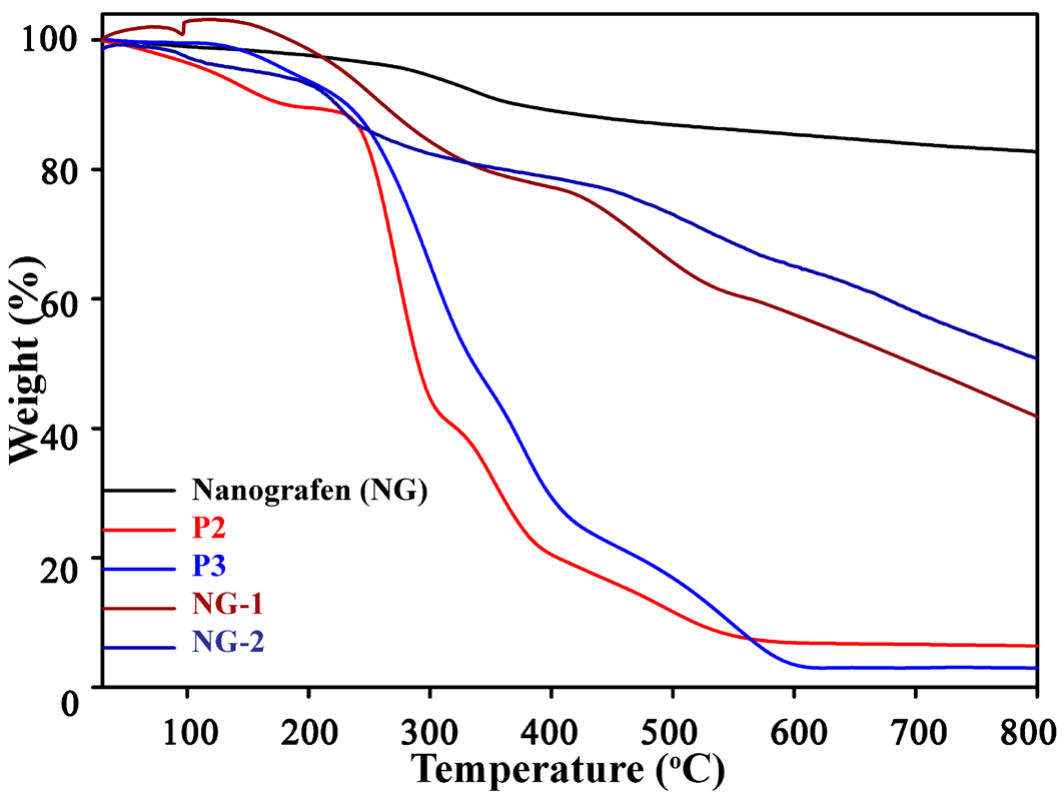
TGA curves of nanographene (NG), P2, P3, NG-1, and NG-2.

**Figure 8:**
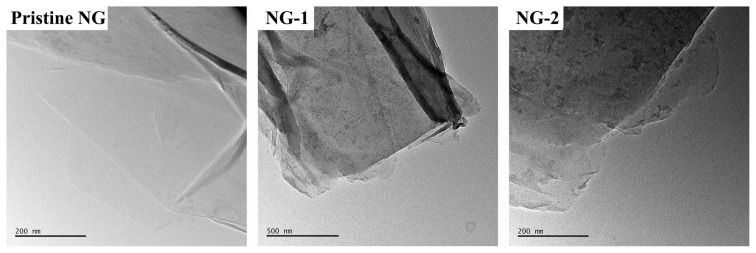
TEM images of pristine and hybrid nanographenes.

30 mg of nanographene (NG) was dispersed in 60 mL of dimethylformamide (DMF) and tetrahydrofuran (THF) mixture (1:5, v/v) in an ultrasonic bath for 10 min. Then, 60 mg P2-hyd was added and left to stir at room temperature for four days. The product was obtained by filtration of dispersion by using a membrane filter (PTFE; pore size: 0.2 μm). Residual solid, namely NG-1, was sonicated with using an excessive amount of ethanol to remove the unloaded copolymer and recovered black solid filtered again until the copolymer could not be detected in the solvent (checked by UV). The obtained black powder was dried under a vacuum. The same procedure was employed by using 60 mg P3-hyd. The obtained material is named NG-2 [31]. Hybrid nanostructures were characterized by TGA, TEM and FTIR measurements. (Figure 7, 8 and S8)

### 2.6. Turbidimetric assay for glycoblock copolymer-lectin aggregation

**Figure 9:**
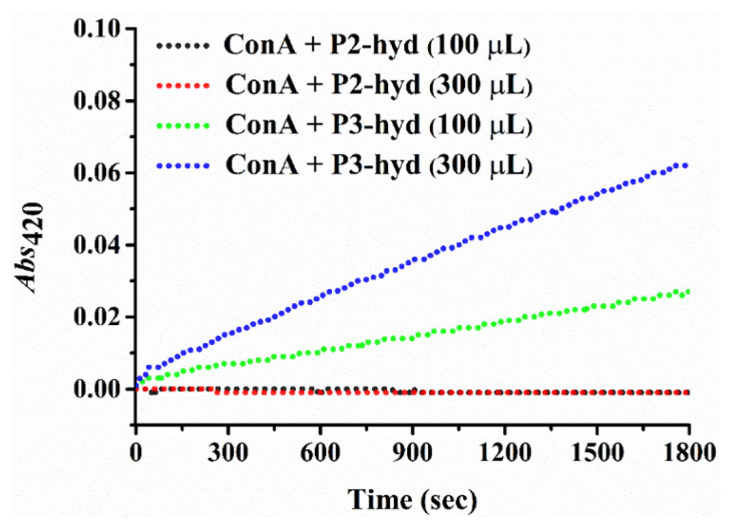
The interaction between ConA with P2-hyd and P3-hyd at different concentrations of the glycopolymer solutions.

To evaluate the lectin binding of glycoblock copolymers, the turbidimetry technique was used [32,33]. Briefly, a stock solution of lectin Concanavalin A (ConA) (0.5 mg mL^−1^) was prepared in HEPES (0.01 M) buffer solution at pH = 7.4. Then, 500 μL of lectin solution was transferred into a quartz cuvette, and the baseline was recorded on UV spectrophotometer. Either 100 μL or 300 μL solution of P(Fruc_1,2_MA-*b*-MMA)-py (P2-hyd) with a concentration of 1 mg mL^−1^ in HEPES was added into the quartz cuvette containing the lectin solution. The sample solution in the cuvette was gently mixed using a glass rod, and, immediately, the absorbance at 420 nm was recorded for 30 min at room temperature. The same procedure was repeated to that of P(Fruc_2,3_MA-*b*-MMA)-py (P3-hyd). As a control experiment, Con A was measured under the same conditions as well as the HEPES buffer solution without any glycoblock copolymer (Figure 9) [34].

## 3. Results and discussion

### 3.1. Synthesis and characterization of glycoblock copolymer

The fructose-based two amphiphilic glycoblock copolymers were used in this study via a three-step synthesis procedure, as shown in Scheme S1. First, a homo-block of MMA was conducted in acetonitrile (ACN) at 70 °C for 6.5 h mediated by pyrene functional 4-cyano-4-((phenylcarbonothioyl)thio) pentanoate (CPADB-py) chain transfer agent together with thermal radical initiator AIBN. Monomer conversion was confirmed by ^1^H NMR spectroscopy. The degree of polymerization (DP) of PMMA-py was found to be 21. This was obtained by comparing the integral of the methylene protons to the pyrene unit at 5.87 ppm to the integral corresponding to the methoxy unit at 3.60 ppm (Figure 2).

After the hydrophobic block was completed, a second block was performed with two fructose-based glycomonomers (*ipr*Fruc_1,2_MA and *ipr*Fruc_2,3_MA). Fructose was used as a starting material by selectively protecting hydroxyl groups of it to form isopropylidene derivatives of fructose methacrylate monomer as described in the literature [27]. Polymerization of *ipr*Fruc_1,2_MA was conducted at 70 °C mediated by PMMA-py as a Macro-RAFT initiator to build the second hydrophilic block together with AIBN in ACN for 8.5 h.

In addition, the same procedure was employed with *ipr*Fruc_2,3_MA. As a result, two glycoblock copolymers were obtained and named P(*ipr*Fruc_1,2_MA-*b*-MMA)-py (P2) and P(*ipr*Fruc_2,3_MA-*b*-MMA)-py (P3). After the purification of glycoblock copolymers via a dissolution-precipitation cycle, the appearance of characteristic –C*H* and –C*H_2_* protons of fructose moiety between 5.25 – 3.75 ppm confirmed the overall structure of the glycoblock copolymers from ^1^H NMR spectra of P2 and P3 (Figure 3 and 4). The mean DPs of P2 and P3 were calculated as 52 and 72, respectively, by comparing the integral of the methylene protons adjacent to the pyrene unit at 5.88 ppm with the fructose moiety at 5.25 – 3.75 ppm.

Following ^1^H NMR analysis, a clear shift in the higher molecular weight distribution was observed. A monomodal chromatogram from GPC measurements without tail or shoulder proved the formation of glycoblock copolymer (Figure S7A–B). Moreover, relatively low dispersity was obtained throughout each polymerization, and final *Đ* values are 1.24 and 1.09.

The low polydispersity index (*Đ*) and *M*_n_ values of P1–P3 were summarized in Table.

In the last step, acid hydrolysis of the isopropylidene groups was conducted with TFA overnight at room temperature. The degree of hydrolysis was estimated by a ^1^H NMR spectra of the glycoblock copolymers. After purification, the disappearance of isopropylidene protons at ~1.2–1.6 ppm confirms the hydrolysis reaction (Figure 5A and 6A). The successful hydrolysis of amphiphilic block glycopolymers was also confirmed with FT-IR and GPC measurements of P2-hyd and P3-hyd (Figures 5B, 6B, and S7B). The disappearance of the C-O absorption band of the isopropylidene groups at 1215 cm^−1^ and the existence of the characteristic broad absorption band of hydroxyl groups at ν = 3350 cm^−1^ verified the successful hydrolysis reaction.

The fabrication of the glycopolymers onto the graphene surface is significant for controlling surface architecture and properties. In this way, P(Fruc_1,2_MA-*b*-MMA)-py (P2-hyd) and P(Fruc_2,3_MA-*b*-MMA)-py (P3-hyd) glycopolymer can be adsorbed onto the graphene surface by electrostatic non-covalent π-π stacking interactions. Biocompatible nanographene hybrids were obtained by the modification of nanographene sheets with pyrene-terminated P2-hyd and P3-hyd via non-covalent π-π stacking interactions.

### 3.2. Synthesis and characterization of glycoblock copolymer decorated nanographene

Thermal decomposition of the NG-1 and NG-2 samples and their precursors were checked by thermogravimetric analysis (TGA). The analysis was performed at temperatures a ranging from room temperature to 800 °C, with a heating rate of 10 °C/min under N_2_ gas flow. TGA analysis showed that the tempering of NG around 400 °C caused 16% weight loss resulting from H_2_O release (dehydration) and thermal decomposition of the organic compounds (CO_2_, CO, and ash formation). 41% and 31% weight loss of NG-1 and NG-2, respectively, were related to the decomposition of glycopolymers fabricated onto the nanographene (Figure 7).

To visualize the apparent difference between the pristine and hybrid nanographene, TEM images of the NG-1 and NG-2 samples were investigated. According to Figure 8, significant differences can be clearly seen after non-covalent functionalization with both glycopolymers.

### 3.3. Turbidimetric assay for glycopolymer-lectin aggregation

To investigate the binding affinity of glycopolymers to ConA, turbidity measurements were performed. Figure 9 shows UV analyses of the formed aggregates. The result demonstrates that the alteration of the amount of the P3-hyd solution increases the turbidity of the solution. Even with the short reaction time and the small amount of lectin, aggregation can be seen, indicating fructose moieties in P3-hyd have a strong ability to bind to ConA. In contrast, no interaction was observed between lectin and P2-hyd solution [35].

## 4. Conclusion

In summary, we have successfully synthesized P(Fruc_1,2_MA-*b*-MMA)-py (P2-hyd) and P(Fruc_2,3_MA-*b*-MMA)-py (P3-hyd) glycopolymers with pyrene functionality. Both glycopolymers showed monomodal distribution, and their polydispersity indexes were lower than 1.15. To overcome the aggregation of nanographene and obtain homogeneous nanoscale materials with highly biologically active groups, nanographene was modified with these fluorescent glycopolymers via π-π stacking interactions. Nanographene hybrid materials were investigated with TGA and TEM analysis. The TGA analysis revealed that the amount of glycopolymers on hybrid materials was found to be ca. 41% for NG-1 and 31% for NG-2 after 4 days of π-π stacking interactions. The investigation of the interactions of the P2-hyd and P3-hyd glycopolymers with ConA, which was performed using time-dependent UV measurements and direct observation of the turbidity of solutions, indicated strong evidence of interaction between ConA and polymer bearing sugar moieties. This π-π stacking attachment approach could be a potent method for producing carefully designed new multivalent glyconanomaterials for use in biomedical applications. Their biocompatible surface can be used in the future to conjugate/encapsulate and release drugs. Furthermore, bioapplications of nanographene-glycopolymer hybrid nanostructures [5], such as in nanomedicine and nanobiosensor, may become a target of research in the near future.

## Supplementary Information



## Figures and Tables

**Table:** GPC Characterization data for pyrene end-capped homopolymer and glycoblock copolymers.

Entry	Polymer	[M]:[CTA]:[I]	*M*_n,GPC_ (kDa)	Đ	Conv.^c^ (%)	*M*_n,NMR_^d^ (kDa)
P1^a^	PMMA_21_-py	50:1:0.125	3.65^a^	1.09^a^	42	2.60
P2^a^	P(*ipr*Fruc_1,2_MA_52_-*b*-MMA_21_)-py	100:1:0.200	16.65^a^	1.24^a^	52	19.70
P3^a^	P(*ipr*Fruc_2,3_MA_72_-*b*-MMA_21_)-py	100:1:0.200	16.46^a^	1.09^a^	72	26.25
P2-hyd^b^	P(Fruc_1,2_MA_52_-*b*-MMA_21_)-py	-	23.15^b^	1.14^b^	-	-
P3-hyd^b^	P(Fruc_2,3_MA_72_-*b*-MMA_21_)-py	-	18.48^b^	1.07^b^	-	-

a Molecular weight (*M*_n_) and *Đ* determined by THF-GPC relative to PS standards.

b Molecular weight (*M*_n_) and *Đ* determined by DMF-GPC.

c Polymerization conversion determined from ^1^H NMR analysis.

d Determined based on the integral ratios of distinct protons by ^1^H NMR in CDCl_3_ (*M*_n,NMR_ = DP determined from ^1^H-NMR X M_W_ of monomer + M_W_ of RAFT agent).
